# Virtual Igor: an analytical phantom for the simulation of the Saint Petersburg brick phantom in arbitrary layouts in MCNP

**DOI:** 10.1007/s00411-021-00939-1

**Published:** 2021-08-28

**Authors:** Oliver Meisenberg

**Affiliations:** Federal Office for Radiation Protection, Ingolstädter Landstr. 1, 85764 Oberschleissheim, Germany

**Keywords:** Analytical phantom, MCNP, Brick phantom, Saint Petersburg brick phantom, Whole-body counting

## Abstract

A computer code called Virtual Igor is presented. The code generates an analytical representation of the Saint Petersburg brick phantom family (Igor, Olga, Irina), which is frequently used for the calibration of whole-body counters, in arbitrary user-defined layouts for the use in the Monte-Carlo radiation transport code MCNP. The computer code reads a file in the ldraw format, which can easily be produced by simple freeware software with graphical user interfaces and which contains the types and coordinates of the bricks. Ldraw files with the canonical layouts of the brick phantom are provided with Virtual Igor. The code determines the positions of (2.75 cm)^3^ segments of the bricks, where 2.75 cm is the smallest length in the layout and, therefore, represents the spacing of the segment lattice. Each segment contains the exact geometry of the respective part of the brick, using cuboid and cylindrical surfaces. The user can define which rod source drill holes of which bricks contain the rod-type radionuclide sources. The method facilitates the comparison of different layouts of the Saint Petersburg brick phantom with each other and with anthropomorphic computational phantoms.

## Introduction

**Fig. 1:**
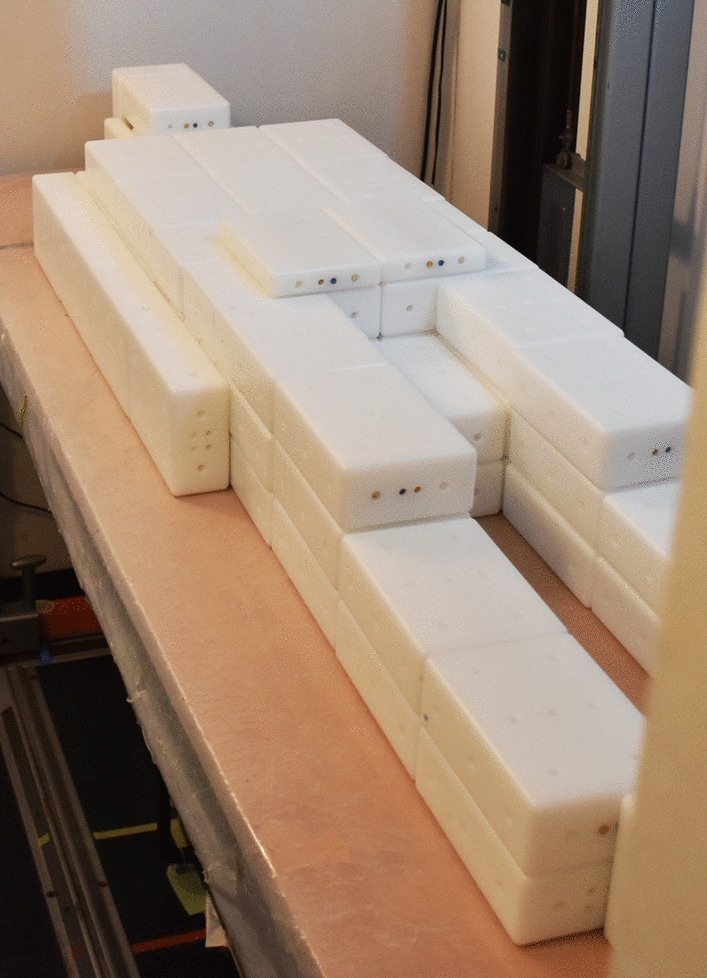
The Saint Petersburg brick phantom in the 70 kg configuration (consisting of 68 full bricks and 4 half bricks) set up in stretcher geometry at a whole-body counting facility

**Fig. 2:**
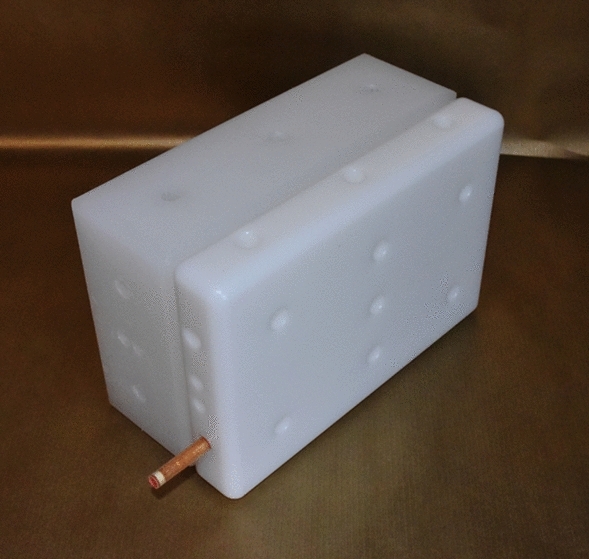
One full brick (left) and one half brick (right) each with four drill holes for radioactive rod sources in the front faces, of which one drill hole inside the half brick is filled with a radioactive rod source. The rod source protrudes from the brick for better visibility wheras during a measurement the source is aligned with the front face of the brick. The drill holes in the other faces are blind holes for metal connectors

The Saint Petersburg brick phantom or universal phantom UPh (also called Igor, Olga or Irina in various countries) is a common resource for the calibration of whole-body counters (ICRU 1992; Kovtun et al. 2000). It consists of bricks made of high-density polyethylene, which can be assembled in different layouts to simulate different physiques and postures of the human body (Fig. 1). Each brick features several drill holes, which can be equipped with rod-shaped sealed radionuclide sources (Fig. 2). Bricks are available with dimensions of 16.5 cm × 11 cm × 5.5 cm (full bricks) and 16.5 cm × 11 cm × 2.75 cm (half bricks); thus, the dimensions of the bricks stand in a ratio of 6:4:2 and 6:4:1, respectively.

It is valuable to simulate such calibration phantoms in Monte-Carlo radiation transport codes like MCNP to reproduce the results of experimental calibrations in computer simulations and to compare such calibrations with calculations using realistic anthropomorphic computational phantoms. The latter makes possible to validate the representativeness of the brick phantoms for a variety of problems in internal dosimetry (such as homogeneous and inhomogeneous spatial distributions of radioactive sources within the body or sources that emit photons with different energies).

The Saint Petersburg brick phantom was implemented and used as a voxel phantom in several studies (Cartemo et al. 2016; de Carlan et al. 2007). However, for some problems, in particular problems where the user wants to modify the position or orientation of parts of the phantom geometry, it might be beneficial to implement the brick phantom as an analytical phantom instead of as a voxel phantom. Analytical phantoms feature the exact geometric shapes of the original (in MCNP making use of the different available types of surfaces and macro-bodies) whereas voxel phantoms are made up of a large number of small cuboid volume elements of homogeneous fill (Goorley 2008), which approximate rounded surfaces and surfaces that do not coincide with the voxel surfaces. For the simulation of the brick phantom, the biggest advantage of an analytical phantom is its greater flexibility for the simulation of arbitrary user-defined layouts (physiques or postures) because the structure of the bricks (such as their cuboid shape and the cylindrical shape of the rod-type radioactive sources) is maintained and each part of the brick (such as the brick itself, each drill hole for the sources, each additional drill hole for metal connectors) is reproduced by a single cell in MCNP. Using an analytical phantom also leads to an easier traceability of the results with respect to different parts of the geometry, such as the attenuation of the radiation compared for different layouts. Additionally, in an analytical phantom the number of volume elements is much smaller than in a voxel phantom, which simplifies the MCNP geometry.

**Fig. 3:**
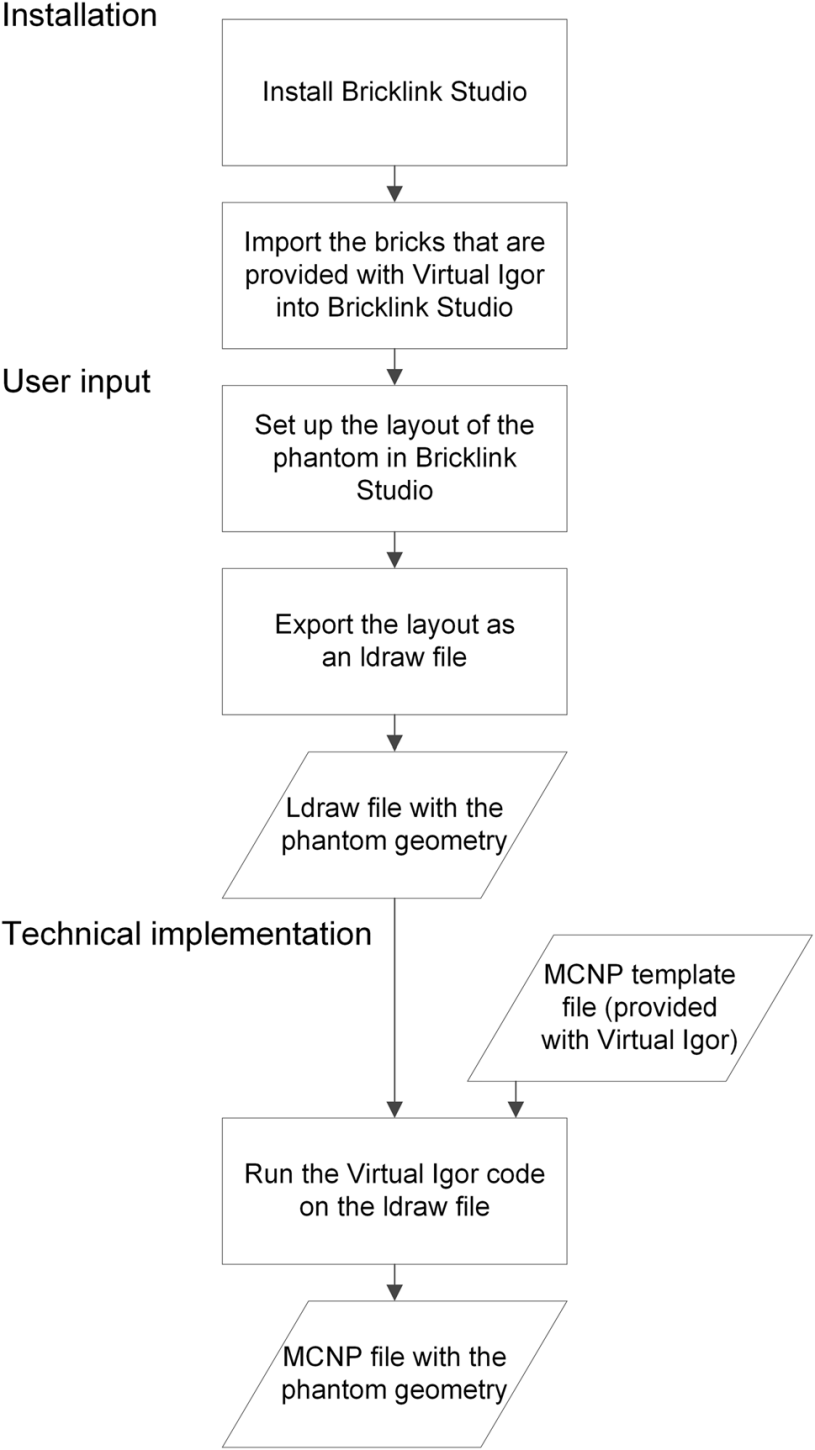
Flowchart of the steps that are required for using Virtual Igor. Cuboids denote user actions, rhomboids denote inputs and outputs

This technical note presents a computer code called Virtual Igor which generates MCNP input files to simulate the geometry and source distribution of the Saint Petersburg brick phantom in any layout (i.e. number, position, and orientation of the bricks) that is defined by the user. Virtual Igor can be downloaded for free and with an open source code together with a technical documentation from www.bfs.de/virtual-igor-en. Figure 3 presents a flowchart of the steps that are required for using Virtual Igor and that are addressed in the following sections.

The code was tested with MCNP version 6.2 (Werner et al. 2018). This was done by setting up a variety of phantoms with different layouts and radioactive sources as well as applying different settings such as the position of the radioactive sources and the length of their active section. From these phantoms MCNP input files were created and the geometry and source-type mesh-tally results were thoroughly examined in the MCNP plotter and in suitable output print tables (such as Table 110 with the starting histories).

## User input

Initially the user of Virtual Igor must define the layout of the brick phantom in a suitable software which is capable of producing files in the ldraw format (LDraw.org Standards Board 2012). Ldraw files contain the 3-dimensional coordinates and orientation of the bricks as a formatted list. An example of such software is Bricklink Studio 2.0 (Lego Bricklink Inc., Irvine, California), which is originally meant for designing Lego models and which is available as freeware online. It features the capability to position bricks on a regular lattice, to detect collisions so that bricks can be placed adjacent to each other, and to stack bricks on top of each other.

Together with the Virtual Igor computer code, files with the contours of the different bricks (full and half bricks) of the Saint Petersburg brick phantom that can be loaded into Bricklink Studio 2.0 are provided for download on the Virtual Igor web page. After importing these files, these bricks can be selected in the Studio software to define layouts of bricks with and without radioactive rod sources. Thus, user-defined layouts of the brick phantom and source distributions within the phantom can be built in the software. Files with the canonical layouts of the brick phantom in the various physiques and postures are provided with Virtual Igor.

## Technical implementation

The generation of the MCNP input file is performed by a Python 3.0 computer code (Python Software Foundation 2020) that works on an MCNP template file provided with Virtual Igor. The MCNP template file contains the geometry of one full brick and one half brick with their physical dimensions, the positions and dimensions of the drill holes for the radioactive rod sources, the structure of the rod sources themselves as well as further parts of the geometry such as the inactive plugs of the rod sources and the drill holes for the metal connectors. The MCNP macro-bodies “rpp” (rectangular parallelepiped) and “rcc” (right circular cylinder) are used for setting up the geometry in the MCNP template file. Subsequently these geometries are divided into 6 × 4 × 2 = 48 segments of the full brick and 6 × 4 = 24 segments of the half brick with dimensions of (2.75 cm)^3^, where 2.75 cm (the thickness of the half brick) is the smallest dimension that occurs in the geometry. Consequently, 2.75 cm is the unit length of the lattice on which the bricks can be positioned. This segmentation is done using universe/fill combinations, which fill the whole full and half brick into the respective number of (2.75 cm)^3^ cells adjacent to each other.

The Python code loads the user-generated ldraw file and reads the position and orientation of each brick. It subsequently determines the position and orientation of each of its 48 or 24 segments on a 3-dimensional lattice of 2.75 cm spacing. The code generates a list of universe numbers referring to the 48 or 24 segments per brick together with the respective rotation matrices. This list is then inserted into the MCNP template file as a fully-specified lattice. Required additional parameters of the lattice (number of lattice elements in the three directions and the physical dimensions of the whole lattice) are inserted into the template file as well so that the output file can immediately be used in MCNP.

The Python code also reads the information which of the drill holes of which bricks are chosen as radioactive rod sources and produces a list of source coordinates and respective MCNP cell numbers. It also produces a list of the correct probabilities of sampling from the respective rod source and the (2.75 cm)^3^ segments over which the rod source is distributed so that the sampling of source-particle coordinates occurs with uniform spatial probability distribution within each single source and with equal overall probability for all rod sources in full bricks and with half that probability in half bricks. This list is inserted into the SDEF source definition of the MCNP template file. Cell rejection and source probability cards are used for this task.

Further parameters of the MCNP simulation such as the energy of photons emitted by the sources or the number of histories to be run need to be adapted in the generated MCNP file by the user. This also holds true for customizations of the phantom geometry such as different brick materials or positions of the rod-source drill holes that differ from those defined in the provided MCNP template file.

## Limitations of the method

Whereas the presented method allows to define virtual replications of the brick phantom in various layouts, it is not possible to deviate from the (2.75 cm)^3^ lattice, e.g. to set up a phantom with legs that approach each other towards the feet.

It is neither possible to automatically set up inflected phantoms with angles different than 90° between different parts of the phantom, e.g. to set up a phantom on an inclined chair. However, such geometries can be generated by running the Python code with ldraw files that contain the single parts of the phantom, which subsequently can be assembled by the user in the MCNP input file with the required angles between them. An example for such a geometry is included in the Virtual Igor documentation.

Virtual Igor is prepared to simulate only one radioactive rod source per brick (not necessarily at equal positions in all bricks). If users want to simulate a phantom with several sources per brick, they can set up and simulate phantoms with one source per brick one after the other and compile the results at the end.

## Conclusion

Virtual Igor allows to prepare MCNP input files for user-defined layouts of the Saint Petersburg brick phantom typically within less than half an hour, of which the construction of the phantom in the Studio software takes the most time. Adaptation of already prepared phantoms typically takes only a few minutes. Therefore, it is easily possible to compare different layouts with each other and with more realistic phantoms such as the ICRP voxel phantoms. This facilitates the optimization of the Saint Petersburg brick phantom (regarding its length, girth, exact position of body parts etc.) with respect to its representativeness compared to the ICRP voxel phantoms. Also, the influence of different postures (such as the lower arm bricks arranged on top of the belly instead of beside it) and physiques (such as a shorter but more obese layout with the same number of bricks) can be studied. As with any other computational phantom, simulations with Virtual Igor phantoms can be conducted with simulated generic detectors as well as with simulated real detectors that are present in whole-body counting facilities; both types of detectors (with their geometries and tally definitions) need to be included in the MCNP file by the user.

It is also possible to simulate the detector response for more sophisticated measurement tasks such as the measurement of neutron or high-energy beta sources, which produce secondary photon radiation and can therefore also be measured with whole-body counters (Meisenberg et al. 2020; Shagina et al. 2006). The simulation of such measurements in MCNP makes possible to gain information of the particle behavior such as the spatial distribution of the photon-radiation production in the calibration phantom, and also to increase the representativeness of the brick phantom for such special applications.

## Data Availability

Not applicable.

## Code availability

The presented computer code is available from www.bfs.de/virtual-igor-en. It is licensed under the GNU Lesser General Public License.
